# Independence of Hot and Cold Executive Function Deficits in High-Functioning Adults with Autism Spectrum Disorder

**DOI:** 10.3389/fnhum.2016.00024

**Published:** 2016-02-05

**Authors:** David L. Zimmerman, Tamara Ownsworth, Analise O'Donovan, Jacqueline Roberts, Matthew J. Gullo

**Affiliations:** ^1^Behavioral Basis of Health, School of Applied Psychology and Menzies Health Institute Queensland, Griffith UniversityMt. Gravatt, QLD, Australia; ^2^School of Education and Professional Studies, Griffith UniversityMt. Gravatt, QLD, Australia; ^3^Centre for Youth Substance Abuse Research, University of QueenslandSt. Lucia, QLD, Australia

**Keywords:** autism spectrum disorder, adults, executive functions, social cognition, neuropsychological assessment

## Abstract

Individuals with autistic spectrum disorder (ASD) display diverse deficits in social, cognitive and behavioral functioning. To date, there has been mixed findings on the profile of executive function deficits for high-functioning adults (IQ > 70) with ASD. A conceptual distinction is commonly made between “cold” and “hot” executive functions. Cold executive functions refer to mechanistic higher-order cognitive operations (e.g., working memory), whereas hot executive functions entail cognitive abilities supported by emotional awareness and social perception (e.g., social cognition). This study aimed to determine the independence of deficits in hot and cold executive functions for high-functioning adults with ASD. Forty-two adults with ASD (64% male, aged 18–66 years) and 40 age and gender matched controls were administered The Awareness of Social Inference Test (TASIT; emotion recognition and social inference), Letter Number Sequencing (working memory) and Hayling Sentence Completion Test (response initiation and suppression). Between-group analyses identified that the ASD group performed significantly worse than matched controls on all measures of cold and hot executive functions (*d* = 0.54 − 1.5). Hierarchical multiple regression analyses revealed that the ASD sample performed more poorly on emotion recognition and social inference tasks than matched controls after controlling for cold executive functions and employment status. The findings also indicated that the ability to recognize emotions and make social inferences was supported by working memory and response initiation and suppression processes. Overall, this study supports the distinction between hot and cold executive function impairments for adults with ASD. Moreover, it advances understanding of higher-order impairments underlying social interaction difficulties for this population which, in turn, may assist with diagnosis and inform intervention programs.

## Introduction

Neurophysiological differences between adults with autism spectrum disorder (ASD) and neurotypical individuals have been well documented (Bauman, 1996; Bauman and Kemper, 2005). Anatomical and functional abnormalities have been identified in the pre-frontal cortex, temporal poles, basal ganglia, and the limbic system, a network of structures underlying executive functions and social cognition (Bauman, 1996; McAlonan et al., 2005; Ashwin et al., 2007; Lieberman, 2007). Yet, there are mixed empirical findings on the profile of executive function deficits experienced by high-functioning adults with ASD, or those with an IQ in the normal range (Boucher et al., 2005; Rajendran et al., 2005; Hill and Bird, 2006; Baez et al., 2012). In particular, it is unclear to what extent social cognition deficits overlap with, or are independent of other executive function deficits. An improved understanding of the higher-order impairments that underlie functional difficulties for individuals with ASD may assist with diagnosis and inform targeted intervention programs.

**Figure 1:**
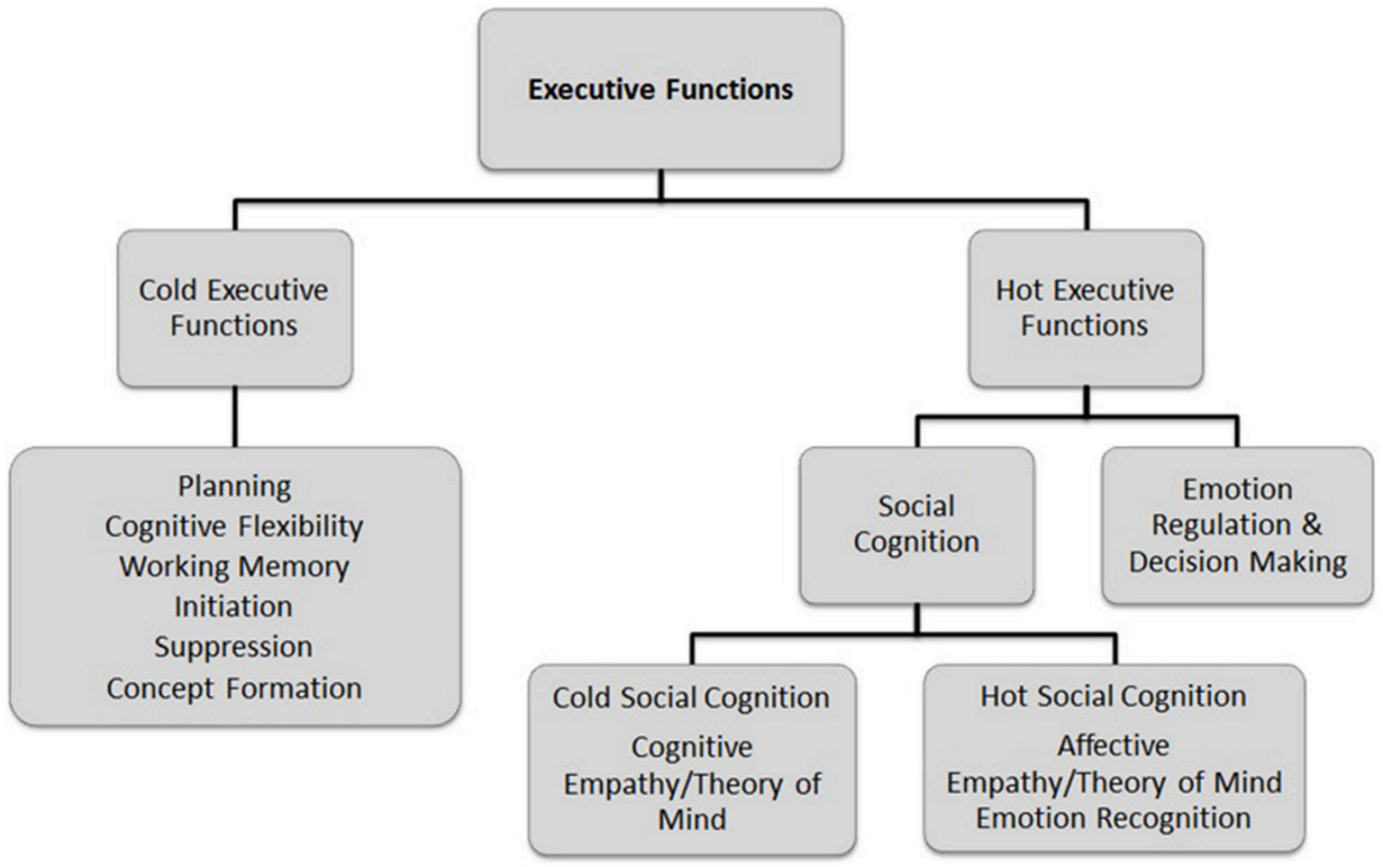
**Framework of executive function processes, adapted from Chan et al. (2008) and McDonald (2013)**.

Described as an umbrella term, executive functions encompass higher-order cognitive processes and behavioral competencies such as planning, cognitive flexibility, social cognition (e.g., empathy and theory of mind [ToM]) and emotion regulation (Chan et al., 2008). These higher-order cognitive functions are mediated by the pre-frontal cortex and provide control and direction to lower-order brain functions (Stuss and Levine, 2002). In the literature, a distinction is commonly made between “cold” and “hot” executive functions (Chan et al., 2008; McDonald, 2013), as outlined in Figure 1.

Cold executive functions are associated with the dorsolateral pre-frontal cortical regions and include planning, cognitive flexibility, working memory, behavioral monitoring, and inhibition (Chan et al., 2008). While there is an extensive body of evidence indicating that individuals with ASD are typically impaired on tests of cold executive functions (Hill, 2004; Ozonoff et al., 2004; Boucher et al., 2005; Rajendran et al., 2005), the findings are mixed in terms of the specific profile of executive dysfunction. For example, deficits in working memory were reported in a number of studies (e.g., Bennetto et al., 1996; Williams et al., 2005; Steele et al., 2007), whereas other studies found little evidence of working memory impairments (Ozonoff and Strayer, 2001; Koshino et al., 2005). Further, there is conflicting evidence regarding impairments in set-shifting or mental flexibility (Diamond and Kirkham, 2005), with deficits reported in one study (Baez et al., 2012), but not in other studies (Kleinhans et al., 2005; Hill and Bird, 2006). There have also been mixed findings for response initiation and suppression, with impairments evident in some studies (Boucher et al., 2005; Hill and Bird, 2006; Johnston et al., 2011) but not others (Abell and Hare, 2005; Baez et al., 2012).

Hot executive functions are mediated by the ventromedial and orbito-frontal cortices, which support behaviors that require emotional awareness and regulation, empathy, and ToM (Chan et al., 2008; McDonald, 2013). Conceptualized as a component of hot executive functions, social cognition is a broad term that encompasses several domains, including: emotion recognition, ToM, central coherence, and empathy (Baez et al., 2012; Baez and Ibanez, 2014). McDonald (2013) distinguished between “hot” and “cold” aspects of social cognition involved in evaluating and interpreting a social situation (see Figure 1). Hot social cognition refers to the processes responsible for emotion perception and identification, such as empathizing with the affective state of another person (e.g., emotional empathy). Cold social cognition involves a more objective perspective, such as thinking about things from another person's point of view (e.g., ToM and cognitive empathy). Essentially, social cognition is a complex set of processes subserving adaptive social interactions, allowing an individual to share cognitive and affective experiences of other people, predict their behavior and communicate effectively (McDonald, 2013). As such, social cognition is recognized as a core domain of impairment for individuals with ASD (Happé et al., 2006; Rajendran and Mitchell, 2007). However, the extent to which social cognition deficits overlap with, or are independent of cold executive function deficits remains unclear.

More generally, there is debate on whether social cognition represents a set of general cognitive abilities applied to social stimuli, or is supported by a dedicated modular cognitive system (Adolphs, 2010). Support for the functional modularity of social cognition is evident from some research on developmental disorders. For example, many high-functioning individuals with ASD demonstrate impairments in social cognition while general intellectual ability is preserved (Boucher et al., 2005). Conversely, individuals with Williams syndrome present with hyper-social behaviors despite impairments in non-social cognitive domains (Adolphs, 1999; Meyer-Lindenberg et al., 2005). Further, neuroimaging research by Castelli et al. (2002) identified that adults with ASD show reduced activation in a network of structures implicated in the attribution of mental states. Although general cognitive abilities (e.g., attention, memory, and language) and cold executive functions most likely play an important role during social interaction, the evaluation and interpretation of emotional and mental states appear to engage unique processes with specific neural substrates (Lieberman, 2007). To investigate whether social cognition operates independently of cold executive functions, reliable, and valid measures of hot executive functions are needed.

Most studies of social cognition in adults with ASD have not distinguished between cold and hot ToM (Baron-Cohen et al., 2003; Uljarevic and Hamilton, 2013). Furthermore, first and second order false belief tasks (Perner and Wimmer, 1985; Perner et al., 1989), such as the Faux Pas Test (Stone et al., 1998) and the Strange Stories Test (Happé, 1994) are text-based and do not closely resemble the demands of everyday social interactions (Mathersul et al., 2013). There is mixed evidence of impairments on the Reading the Mind in the Eyes Test (RMET; Baron-Cohen et al., 1997, 2001; Couture et al., 2010; Baez et al., 2012). The RMET is a static measure of ToM that can be solved using basic and general matching strategies to correctly pair depicted eyes and emotions. Although the RMET has been used extensively to assess ToM in people with ASD, the test's ecological validity has been questioned (Jarrold et al., 2000; Johnston et al., 2008).

McDonald et al. (2003, 2006) developed The Awareness of Social Inference Test (TASIT), a dynamic audio-visual assessment, to more closely approximate the social cognition skills required during social interaction. TASIT assesses basic emotion recognition (TASIT Part 1) and cognitive and affective ToM (Parts 2 and 3; TASIT; McDonald et al., 2003). Mathersul et al. (2013) used TASIT Parts 2 and 3 to compare the social cognition performance of adults with ASD to matched controls. Individuals with ASD were found to demonstrate impairments in understanding the beliefs, intentions and meaning of non-literal expressions (i.e., lies and/or sarcasm) relative to controls. However, their ability to accurately interpret social interactions that involved a sincere exchange was consistent with controls.

Performance on social cognition measures such as TASIT is likely to be influenced by both hot and cold executive functions (McDonald et al., 2006). Yet, Baez et al. (2012) found that after controlling for cognitive flexibility (i.e., Switching Design Fluency), adults with ASD performed more poorly than controls on the emotion recognition test of TASIT (Part 1; McDonald et al., 2003), and emotional and cognitive inference aspects of ToM (FPT). Therefore, deficits in cognitive flexibility could not account for the impaired performance of the ASD group on tests of hot executive function relative to controls. To interpret dynamic social interactions, multiple sources of information need to be held online (i.e., working memory) whilst integrating relevant cues (e.g., facial expression, body language, linguistic content, and context) in order to understand the meaning of the interaction and provide an appropriate and timely response. Accordingly, deficits in working memory and response initiation and suppression potentially contribute to social cognition impairments for individuals with ASD, although this has yet to be investigated. Such research would advance understanding of the higher-order cognitive deficits underlying difficulties with social interaction for this population.

The broad objective of the present study was to determine the independence of deficits in hot executive function and cold executive function for high-functioning adults with ASD. Due to their relevance to dynamic social cognition tasks, the cold executive function domains of focus were working memory and response initiation and suppression. The first study aim was to investigate impairments in hot and cold executive functions in high-functioning adults with ASD relative to matched controls. It was hypothesized that individuals with ASD would perform more poorly than controls on tests of cold and hot executive functions. Further, we sought to determine whether differences in hot executive function (TASIT) between participants with ASD and controls were significant after controlling for cold executive functions (i.e., working memory and response initiation and suppression).

## Methodology

### Design

Control participants were matched to high-functioning individuals with ASD on the basis of gender, age, and years of education. Based on studies of executive functions in the adult ASD population (Hill and Bird, 2006; Baez et al., 2012; Mathersul et al., 2013), which demonstrated medium to large effect sizes (control group comparisons), the sample size requirement was determined using G^*^Power 3.1.9.2 (Faul et al., 2009). With power of 0.80 and alpha set at 0.05 (one-tailed), power analyses indicated that a sample of approximately 40 participants per group was required to detect significant between group differences.

### Participants

The broader sample consisted of 42 high-functioning adults with ASD and 40 matched controls. Participants with ASD were recruited via convenience sampling from ASD support services and clinics within a major metropolitan area. The directors and staff were initially contacted regarding the study and they circulated advertisements for the study in person and through online forums. The principal investigator (DZ) additionally presented an overview of the study at several ASD support group meetings.

All participants were screened to determine eligibility for participation, meeting the following criteria: (1) formal diagnosis of ASD and/or self-reported history of longstanding difficulties in social interaction and communication and a restricted range of behaviors and/or interests as reflected by scores equal to or greater than 77 on the Ritvo Autism Asperger's Diagnostic Scale—Revised (RAADS; Ritvo et al., 2011); (2) aged 18–70 years; (3) adequate understanding of spoken and written English; (4) not currently experiencing a comorbid psychotic disorder; (5) no history of a serious neurological or medical condition (e.g., traumatic brain injury); and (6) absence of suspected literacy difficulties on a validated reading test. Further, all participants were administered a test of non-verbal IQ (Matrix Reasoning) to determine whether their estimated IQ was in the normal range (i.e., ≥70). This is in line with previous ASD research that has used IQ scores ≥ = 70 to indicate high-functioning status (Baron-Cohen, 2000; Howlin, 2003). The mean estimated non-verbal IQ for the ASD sample was 102.65 (12.65), with scores ranging from 75 to 131; thus, all participants were considered to be high-functioning in terms of their estimated non-verbal IQ.

The control group participants were matched as closely as possible to the ASD group on gender, age, and years of education. Control participants were recruited from a university and the general community. As shown in Table 1, participants in both groups were predominantly male (ASD group = 64.3%; Control group = 57.5%), with an average age of 34.02 years for the ASD sample and 33.23 years for the control sample. Although the mean years of education were higher in the control group, there were no significant differences between the groups on any of the matching variables.

**Table 1:** **Demographic information and ASD symptom severity for the ASD and matched control groups**.

**Demographic data**	***N*/Mean (*SD*/%), range**			
	**ASD group (*n* = 42)**	**Control group (*n* = 40)**	***t*/χ^2^**	***p***
Age (years)	34.02 (12.42), 18–66	33.23 (12.41), 18–62	–	NS
**GENDER**				
Male	27 (64.3%)	23 (57.5%)	–	NS
Female	15 (35.7%)	17 (42.5%)	–	NS
Years of education	14.05 (2.24)	15.18 (2.92)	–	NS
**ETHNICITY**				
European/Caucasian	40 (95.2%)	30 (75%)		
Asian	–	10 (25%)		
A/TSI^a^	2 (4.8%)	–		
**EMPLOYMENT STATUS**				
Employed^b^	21 (50%)	27 (67.5%)		
Student	7 (16.6%)	11 (27.5%)		
Volunteer	2 (4.8%)	1 (2.5%)		
Unemployed	12 (28.6%)	1 (2.5%)		
**FRIENDSHIP STATUS**				
1 > close friends	19 (45.2%)	40 (100%)		
1 > friends, not close	9 (21.4%)	–		
Group friends only	6 (14.3%)	–		
No close friends	8 (19.1%)	–		
**RELATIONSHIP STATUS**				
Married/De facto	18 (42.9%)	29 (72.5%)		
single	24 (57.1%)	11 (27.5%)		
RAADS-R score^c^	139.05 (36.15), 79–208	37.05 (12.10), 10–58	17.07	*p* < 0.001
ASD formal diagnosis	31 (73.8%)	N/A		
Matrix reasoning	102.38 (12.65), 75–131	N/A		
WIAT reading subtest^d^	107.64 (9.81), 78–119	N/A		

*NS, Not significant*.

a *A/TSI, Aboriginal/Torres Strait Islander*.

b *Employed or Self-Employed*.

c *RAADS-R, Ritvo Autism Asperger Diagnostic Scale-Revised*.

d *WIAT, Wechsler Individual Achievement Test*.

As shown in Table 1, most participants in both groups identified as European/Caucasian. Approximately 67% of individuals in the ASD group were employed or in higher education, as compared to 95% of control participants. In terms of friendship status, 100% of the control group identified having one or more close friends compared to 45.2% of individuals in the ASD group. A higher proportion of individuals in the control group (72.5%) were in a relationship compared to those with ASD (42.9%). All 42 participants identified a history of longstanding ASD symptomatology according to Diagnostic and Statistical Manual of Mental Disorders-Text Revision (DSM-IV-TR) criteria, with scores on the RAADS-R equal to or greater than the established clinical cut-off (i.e., ≥77; range: 79–205) (American Psychiatric Association, 2000; Ritvo et al., 2011). Seventy-four percent also reported a prior formal diagnosis of either ASD or Asperger's syndrome. There was no evidence of clinically significant literacy difficulties for the ASD sample, with a mean scaled score of 107.64 (12.65), and scores ranging from 78 to 119 on a standardized reading test (see Materials).

Data on adaptive functioning and psychological status (see Materials) were collected as part of a broader research project focusing on psychosocial outcomes of adults with ASD. In terms of adaptive functioning (Table 2), most participants with ASD were classified as having Good (40.5%) or Fair/Poor (38.1%) functioning, whereas 21.4% were classified as having Very Good functioning. There were no individuals classified as having Very Poor adaptive functioning. A measure of self-reported psychological status (see Materials) indicated that approximately half of the sample (i.e., 43–48%) were in the normal range for depression, anxiety and stress. However, severe or extremely severe mood symptoms were reported by 41% of the sample for depression and 29% for both anxiety and stress.

**Table 2:** **Descriptive statistics for current mental health and adaptive functioning for the ASD group (***n*** = 42)**.

**Test**	**Mean (*SD*)/Range/Frequency (%)**	**Frequency (%)**		
		**Normal**	**Mild/Moderate**	**Severe/Extremely severe**
**MENTAL HEALTH**				
DASS—Depression	8.86 (9.05), 0–28	19 (45%)	6 (14%)	17 (41%)
DASS—Anxiety	5.10 (5.83), 0–24	18 (43%)	12 (28.5%)	12 (28.5%)
DASS—Stress	8.57 (7.41), 0–30	20 (47.5%)	10 (24%)	12 (28.5%)
**ADAPTIVE FUNCTIONING**				
Very good	9 (21.4%)	–	–	–
Good	17 (40.5%)	–	–	–
Fair/Poor	16 (38.1%)	–	–	–
Very Poor	–			

DASS, Depression, Anxiety and Stress Scales.

### Materials

#### Screening assessments

##### Ritvo Autism Asperger's diagnostic scale—revised (RAADS; Ritvo et al., 2011)

The RAADS-R is a self-report measure based on the DSM-IV-TR and International classification of diseases: Tenth revision (ICD-10 criteria) which is used to assist in the diagnosis of adults with ASD. The scale consists of 80-items (i.e., 64 symptom and 16 non-symptom based responses). Each question is rated on a 4-point Likert scale in order of severity ranging from “True now and when I was young” = 3 to “Never true” = 0. To minimize response bias, the 16 non-symptom based responses are reverse scored and are indicated by an asterisk beside each item. A total score of ≥77 out of a maximum score of 240 is indicative of an ASD diagnosis (Ritvo et al., 2011). The RAADS-R has demonstrated excellent test re-test reliability (*r* = 0.99) and sensitivity (97–100%) and specificity (100%; Ritvo et al., 2008, 2011).

##### Matrix reasoning: Wechsler abbreviated scale of intelligence (WASI; Wechsler, 1999)

The WASI measures estimated verbal and non-verbal intelligence (i.e., Matrix Reasoning), and can yield a Full Scale intelligence quotient (IQ). Due to possible language difficulties impacting estimated IQ, only the Matrix Reasoning subtest was administered as a measure of estimated non-verbal IQ to help determine eligibility for those in the ASD group (i.e., IQ ≥70 based on T-score equivalent of >30).

##### Wechsler individual achievement test-third edition (WIAT III; Wechsler, 2009)

The Word Reading subtest of the WIAT was administered as a screening assessment of literacy difficulties for high-functioning individuals with ASD. Individuals read from a list of 35 words, with each correctly pronounced word scored as one point and converted to a scaled score. In the present study, no participant with ASD performed in the range indicating suspected literacy difficulties (i.e., scores <70; Wechsler, 2009).

##### Depression, anxiety, and stress scales-21 (DASS-21; Lovibond and Lovibond, 1995)

The DASS-21 comprises three 7-item subscales that assess the negative emotional states of depression, anxiety and stress and is based on the original 42-item version. Respondents are asked to rate the extent to which each item applied to them over the past week on a 4-point Likert scale, whereby higher scores indicate greater emotional distress. Scores on each 7-item scale are doubled to enable the following clinical cut-offs to be applied: Depression 0–8 (normal), 9–13 (mild), 14–22 (moderate), 24–36 (severe), and >36 (very severe); Anxiety 0–5 (normal), 6–8 (mild), 9–15 (moderate), 16–26 (severe), and >26 (very severe); Stress 0–14 (normal), 15–18 (mild), 19–26 (moderate), 27–37 (severe), and >37 (very severe; Lovibond and Lovibond, 1995). The DASS was used in the present study to provide descriptive information on the current mental health status for the ASD group.

##### Adaptive functioning

For descriptive purposes for the ASD sample, a composite measure of overall adaptive functioning was derived from key demographic information (i.e., employment, relationship status, independence, and friendships), with low scores indicating better outcomes. Outcome ratings were determined using a scale adapted from Howlin et al. (2004) and Lotter (1978): Very Good = employed/studying, one or more close friends, high level of independence, in a relationship (total scores 0–2); Good = working or studying in some capacity; requiring some degree of support in daily living; some friends/acquaintances (total scores 3–5); Fair/Poor = has some degree of independence; requiring special residential provision/high level of support; no friends outside of residence; voluntary work (total scores 6–11); Very Poor = needing high-level hospital based or institutional care; no friends; no autonomy or independence (total score ≥11).

#### Measures of cold executive functions

##### Hayling sentence completion test (Hayling; Burgess and Shallice, 1997)

The Hayling Test is designed to assess problems with response initiation and suppression. In the first part, the test administrator reads aloud 15 incomplete sentences that the respondent is required to complete with a word that would make the sentence meaningful. The second part is comprised of an additional 15 incomplete sentences that the individual needs to complete with a word that does not fit the context. Four scores are obtained: time taken for part one and two (response initiation [Box A and Box B]), errors for part two (response suppression and concept formation [Box C]) and an overall score (total of Box A, B, and C scores). All response latency and error scores are converted to scaled scores that range from 1 (impaired) to 10 (very superior), with a score of 6 indicating average ability. Burgess and Shallice (1997) report test-retest reliability coefficients ranging from *r* = 0.52 (total errors) to *r* = 0.78 (Part 2, time) over an interval of 2 days to 4 weeks. The Hayling Test has good convergent validity with other measures of response inhibition (e.g., Stroop Inference Test and Color Trails Test; Stroop, 1935; D'Elia et al., 1996) and is sensitive to executive impairment in ASD (Boucher et al., 2005; Hill and Bird, 2006).

##### Letter number sequencing (LNS; Wechsler, 2008)

LNS is a subtest of the Wechsler Adult Intelligence Scale-Fourth Edition (WAIS-IV) designed to assess working memory. During this test, the examiner reads a string of numbers and letters (e.g., C-5-A-1) of increasing length across trials. Test-takers are required to say numbers first in ascending order and letters second in alphabetical order (e.g., 1-5-A-C). Correct responses for each item are totalled across trials and range from 0 to 30 with lower scores indicating poorer working memory. Scaled scores are derived using aged-based normative data (Wechsler, 2008). Studies have reported adequate test-retest reliability (*r* = 0.75) for the LNS and good ecological validity in the context of predicting functional status in a clinical population (Nuechterlein et al., 2008).

#### Measures of hot executive functions

##### The awareness of social-inference test-revised (TASIT; McDonald et al., 2006)

TASIT is a dynamic audio-visual based assessment of social cognition. Comprised of a series of 15–60 second video vignettes, TASIT requires participants to implicitly encode and integrate contextual information to understand the social situation in three conditions: (1) Emotion Evaluation; (2) Social Inference–Minimal (SI-M); and (3) Social Inference–Enriched (SI-E). TASIT 2 and 3 assess simple and complex ToM judgments (Mathersul et al., 2013). For emotion evaluation (part 1), there are 28 scenes for which the respondent is required to identify an actor's emotional state by choosing from one of six basic emotions: happy, sad, angry, anxious, surprised, and revolted. When the actor is not exhibiting any particular emotion, the participant is advised to select a neutral response. Each correct response is allocated one point and then summed for a total score, ranging from 0 to 28. In addition, the seven response options can be classified into either positive (i.e., happy, surprised, and neutral) or negative (i.e., sad, angry, anxious, and revolted) emotions (Flanagan et al., 2002).

In Part 2, the SI-M subtest assesses the ability to determine the meaning and intention of a speaker's dialogue, emotional expression and other paralinguistic cues with minimal context. Comprised of 15 short video scenes of social interactions, the participant responds to four separate questions related to what they think the key actor was *doing, saying, thinking*, and *feeling* toward another person. The respondent answers in three possible ways: yes, no or don't know. There are five scenes in which the actors are *sincere* (i.e., the actors thoughts and feelings are congruent with the words they use), five scenes in which the actors are *sarcastic* using paralinguistic cues (e.g., tone of voice) and fives scenes in which *paradoxical sarcasm* is utilized. Each correct response is allocated one point and then summed for the question type (i.e., do, say, think, and feel) to yield a total score ranging from 0 to 60.

Part 3 (SI-E) incorporates the same response format as SI-M, but examines an individual's ability to understand social inferences within an enriched context. For the 16 scenes, half the videos depict an actor telling a *lie* while the remaining scenes involve *sarcasm*. In addition, eight scenes use either a visual or verbal cue to reveal the true state of affairs or the true beliefs of the speaker. Each correct response is allocated one point and scores are summed for the question type (i.e., do, say, think, and feel) and the number of items to calculate a total score ranging from 0 to 64. TASIT 3 is proposed to assess second-order or advanced ToM, whereby the respondent must infer the thoughts of one actor toward another actor (Baron-Cohen, 1995).

TASIT has been widely validated as a measure of social cognition in different clinical populations, including traumatic brain injury (McDonald et al., 2003; McDonald and Flanagan, 2004; McDonald and Saunders, 2005), schizophrenia (Kern et al., 2009; Sparks et al., 2010; Chung et al., 2011) and dementia (Kipps et al., 2009). It has also demonstrated validity as a measure of social cognition for high-functioning adults with ASD (Mathersul et al., 2013). TASIT has sound psychometric properties with good test-retest reliability over a period of 5–26 weeks (*r* = 0.74 − 0.88) and evidence of convergent and discriminant validity (McDonald et al., 2006; McDonald, 2012).

#### Procedure

Ethical clearance was granted by the Griffith University Human Research Ethics Committee (protocol number PSY/28/13/HREC) and the study was conducted in accordance with the National Statement on Ethical Conduct in Human Research. The assessment process was conducted over two phases: (1) demographic survey and the RAADS-R were completed online via a web-link based survey emailed to individuals; and (2) face-to-face administration of a cognitive test battery. Participants in the ASD group received a $20 gift voucher for their participation. The control group participants were recruited via the researchers' social networks and the Griffith University psychology subject pool whereby individuals received course credit for their participation.

In the first assessment phase, participants provided their informed consent followed by questions relating to their demographic and social functioning status using LimeSurvey, a free open source software tool (Schmitz, 2015). For the face-to-face assessment, the Hayling Sentence Completion Test, LNS, and TASIT were administered in a quiet room. To ensure that results from the cognitive test battery were not confounded by order effects (Shum et al., 2006), each test was assigned a number and the sequence was randomized to determine the order of administration for each participant.

#### Data analysis

The Statistical Package for Social Sciences (SPSS) Version 22 for Windows was utilized for all analyses and data screening procedures were conducted according to guidelines by Tabachnick and Fidell (2007). Data were examined for entry errors and missing values. The descriptive data revealed plausible ranges, means and standard deviations for all variables. Neuropsychological performance was classified into the categories of Normal performance and Mild/Moderate (i.e., ≥ − 1 *SD*) and Severe (i.e., ≥ − 2 *SD*) impairment based on the normative data for each test.

Frequency analysis revealed no missing data for the ASD and control group. As it was intended to conduct analyses on group data, univariate outliers, and assumptions of normality were examined separately for the control and ASD groups. Examination of total scores revealed a violation of normality for TASIT 2. This was successfully transformed to correct the negatively skewed data. There were no univariate outliers in the total scores of executive function measures. Inspection of subscales within TASIT 1–3 and Hayling revealed numerous violations of normality and univariate outliers (i.e., z > ± 3.29) for which transformations were unable to correct (note: this was the case for subscales but not total scores). Thus, non-parametric tests (Mann Whitney *U*-tests) were conducted to test the hypothesis regarding differences between the ASD and control groups on measures of executive function. Holm's (1979) step-down procedure was employed to control the Familywise error rate. This method involves ordering *p* values from lowest to highest and cumulatively adding each value until 0.05 is reached. The null hypothesis is rejected for all values less than the cumulative 0.05 total (Holland and Copenhaver, 1988; Aickin and Gensler, 1996).

Three sets of hierarchical multiple regression analyses were conducted with TASIT 1–3 total scores as the outcome variables to determine whether between-group differences in social cognition were significant after controlling for potential covariates and cold executive functions. There were no significant associations between TASIT performance and age, gender and education. However, independent *t*-tests revealed significant between group differences on TASIT 1 and 2 total scores according to employment status (dummy coded). Employment status (for TASIT 1 and 2 only) and scores on Hayling and LNS and were entered in the first step of the regression and ASD status (dummy coded) was entered in the second step. There were no violations of multivariate assumptions for these variables.

## Results

### Comparison of ASD and control groups on measures of executive functioning

Descriptive statistics were generated for TASIT parts 1–3, LNS and Hayling (see Table 3). Based on TASIT norms, the majority of participants (i.e., >50%) in the control and ASD groups were in the normal range for emotion evaluation (Part 1), social inference-minimal (Part 2), and social inference-enriched (Part 3). However, a greater proportion of participants in the ASD group (38–45%) was classified as having either mild/moderate or severe impairment than the control group (0–7.5%) on all parts of TASIT (see Table 3).

**Table 3:** **Executive function performance and classification of impairment for the ASD and control groups**.

**Test**	**Scoring**	**Median/*n*(%)**		
		**ASD (*n* = 42)**	**Controls (*n* = 40)**	***U/z* (Effect size)**
**TASIT**				
EE (part 1)	Raw scores	23	26	257.5/-5.46 (0.60)^*^^b^
Normal	Normative	25 (59.5%)	40 (100%)	
Mild/Moderate	descriptions^a^	10 (23.8%)	–	
Severe		7 (16.7%)	–	
Happy	Raw scores	3	4	547/−3.09 (0.34)^*^
Surprised		4	4	612.5/−2.77 (0.31)^*^
Neutral		3	3	581.5/−2.65 (0.29)^*^
Sad		3	4	637/−2.15 (0.24)^*^
Angry		3	4	402.5/−4.62 (0.51)^*^
Anxious		4	4	654/−2.04
Revolted		4	4	693)/1.79
SI-M (part 2)	Raw scores	50 (319)	56	−4.84 (0.53)^*^
Normal	Normative	23 (54.8%)	37 (92.5%)	
Mild/Moderate	descriptions	7 (16.7%)	3 (7.5%)	
Severe		12 (28.6%)	–	
Do	Raw scores	13	14	441.5/−3.83 (0.42)^*^
Say		12.5	14	365/−4.53 (0.50)^*^
Think		12	14	385/−4.28 (0.47)^*^
Feel		13	14	336.5/−4.80 (0.53)^*^
SI-E (part 3)	Raw scores	52	58	337.5/−4.67 (0.52)^*^
Normal	Normative	26 (61.9%)	37 (92.5%)	
Mild/Moderate	description	6 (14.3%)	3 (7.5%)	
Severe		10 (23.8%)	–	
Do	Raw scores	14	14	636.5/−1.93
Say		13	14	573.5/−2.51 (0.28)^*^
Think		13	14	728/−1.07
Feel		13	13.5	577.5/−2.49 (0.27)^*^
LNS	Scaled scores	10	11	526/−2.95 (0.33)^*^
Hayling	Scaled scores			
Box A		5	6	460/−3.83 (0.42)^*^
Box B		6	6	446/−4.38 (0.48)^*^
Box C		7	7	601.5/−2.33 (0.26)^*^
Total Score^c^		17	19	365.5/−4.48 (0.49)^*^

* *p < 0.05; EE, Emotion Evaluation; LNS, Letter Number Sequencing; SI-M, Social Inference-Minimal; SI-E, Social Inference-Enriched; TASIT, The Awareness of Social Inference Test*.

a *Classification relative to TASIT norms: Mild/Moderate ≥ − 1 SD to < −2 SD; Severe ≥ − 2 SD*.

b *Significance values adjusted using Holm's procedure*.

c *Total Scaled Scores from Box A, B, and C*.

As shown in Table 3, significant between group differences were evident on most TASIT subscales and LNS and Hayling scores. The medians indicated that the control group consistently outperformed the ASD group on these measures. Effect sizes (*r*) were manually calculated using *z* scores as recommended by Pallant (2013). According to Cohen's (1988) criteria, effect sizes ranged from small to large (*r* = 0.26 − 0.60).

For TASIT Part 1, the seven response options are classified into either positive (i.e., happy, surprised and neutral) or negative (i.e., sad, angry, anxious, and revolted) emotions. Mann-Whitney U tests revealed a significant overall difference in the ability to identify positive emotions between the ASD group (*Md* = 10) and the control group (*Md* = 11), *U* = 382.5, *z* = −4.38, *p* < 0.001, *r* = 0.48). In addition, there was a significant overall difference in detecting negative emotions between the ASD group (*Md* = 13) and the control group (*Md* = 15), *U* = 299, *z* = −5.15, *p* < 0.001, *r* = 0.60). The medians suggested that the control group were significantly better at identifying both positive and negative emotions than the ASD group.

For TASIT Part 2, vignettes are categorized according to whether the social interaction was sincere or involved simple sarcasm or paradoxical sarcasm. Mann-Whitney U tests revealed a significant difference in the ability to interpret sincere interactions between the ASD group (*Md* = 16) and control group (*Md* = 19), *U* = 541.5, *z* = −2.80, *p* = 0.005, *r* = 0.31). There was also a significant difference in the ability to detect simple sarcasm between the ASD group (*Md* = 16.5) and control group (*Md* = 20), *U* = 365, *z* = −4.55, *p* < 0.001, *r* = 0.50). Finally, there was a significant difference in the ability to identify paradoxical sarcasm between the ASD group (*Md* = 18.5) and control group (*Md* = 20), *U* = 556.5, *z* = −2.77, *p* = 0.006, *r* = 0.31). The median scores indicated that the controls were significantly more accurate than participants with ASD group in making social inferences with minimal context.

For TASIT Part 3, vignettes related to situations involving either lies or sarcasm. Mann-Whitney U tests revealed a significant difference in the ability to understand social inferences involving lies between the ASD group (*Md* = 26) and control group (*Md* = 29), *U* = 459.5, *z* = −3.55, *p* < 0.001, *r* = 0.39). Further, there was a significant difference in the ability to detect sarcasm between the ASD group (*Md* = 26) and control group (*Md* = 29), *U* = 404.5, *z* = −4.06, *p* < 0.001, *r* = 0.45). The median scores indicated that the control group demonstrated a greater ability to make social inferences in situations with enriched information than participants with ASD.

Given the generalized pattern of impairment across hot and cold executive functions demonstrated by the ASD group relative to controls, it was relevant to examine whether the between-group differences in hot executive function were significant after controlling for performance on tests of cold executive function.

### Independence of impairments in hot and cold executive functions

Table 4 displays the descriptive statistics and correlations for TASIT, LNS and Hayling for the combined control and ASD sample. The measures of executive functioning were significantly inter-related, with medium to large association (*r* = 0.42 − 0.48) between TASIT and Hayling, and small to medium associations between TASIT and LNS (*r* = 0.25 − 0.34).

**Table 4:** **Means, standard deviations and correlations between TASIT part 1–3, Hayling and LNS (***n*** = 82)**.

	**Mean**	***SD***	**TASIT Part 2**	**TASIT Part 3**	**Hayling**	**LNS**
TASIT Part 1	24.49	2.21	0.51^**^	0.49^**^	0.48^**^	0.34^*^
TASIT Part 2	52.20	6.29		0.69^**^	0.47^**^	0.25^*^
TASIT Part 3	54.22	6.73			0.42^**^	0.29^*^
Hayling^a^	17.37	3.22				0.23^*^
LNS^b^	10.63	2.82				

* *p < 0.05*,

** *p < 0.01*.

a *Total scaled scores from Box A, B, and C*.

b *Letter Number Sequencing scaled score; TASIT, The Awareness of Social Inference Test*.

As shown in Table 5, Hayling, LNS and employment status initially explained 36.8% of the variance in TASIT Part 1 [*F*_(3, 78)_ = 15.11, *p* < 0.001]. When ASD status was entered in Step 2, the total variance explained significantly increased to 46.3%, *F*_(4, 77)_ = 16.34, *p* < 0.001 (adjusted *R*^2^ = 0.43). ASD status explained an additional 9.2% of the variance in TASIT Part 1 scores, *F* change_(1, 77)_ = 13.03, *p* < 0.01. In the final model, ASD status (*sr*^2^ = 0.09), Hayling (*sr*^2^ = 0.04) and LNS (*sr*^2^ = 0.03) were all significant predictors. Therefore, the ASD group demonstrated deficits in emotional evaluation relative to controls that were independent of impairments in cold executive function and employment status.

**Table 5:** **Hierarchical multiple regression analysis of the relationship between ASD status and TASIT part 1 (emotion evaluation) controlling for Hayling, LNS and employment status (*n* = 82)**.

**Variable**	**B**	**β**	***sr*^2^**	***p***	**95% CI**
Constant	17.55				[14.93, 20.17]
**STEP 1**					
Hayling	0.28	0.39	0.13	<0.001^***^	[0.14, 0.42]
LNS	0.20	0.24	0.05	0.012^*^	[0.05, 0.36]
Employment^a^	−1.3	−0.21	0.04	0.024^*^	[−2.47, −0.18]
**STEP 2**					
Hayling	0.18	0.25	0.04	0.013^**^	[0.04, 0.32]
LNS	0.15	0.19	0.03	0.04^*^	[0.01, 0.30]
Employment	−0.76	−0.12	0.02	0.17	[−1.87, 0.34]
ASD status^b^	1.80	0.37	0.09	0.001^***^	[0.81, 2.80]

* *p < 0.05*,

** *p < 0.01*,

*** *p < 0.001*.

a *Employment: 1, Employed/Student; 2, Unemployed*.

b *ASD status: 1, ASD; 2, Control*.

For TASIT 2 (see Table 6), Hayling, LNS and employment status initially explained 25.7% of the variance in performance [*F*_(3, 78)_ = 9.00, *p* < 0.001). When ASD status was entered in Step 2, the total variance explained increased significantly to 34.6%, *F*_(4, 77)_ = 8.89, *p* < 0.001 (adjusted *R*^2^ = 0.31). ASD status explained an additional 8.9% of the variance in TASIT Part 2 scores, *F* change_(1, 77)_ = 10.43, *p* = 0.002. In the final model, only Hayling (*sr*^2^ = 0.04) and ASD status (*sr*^2^ = 0.09) were significant predictors. Thus, individuals with ASD demonstrated deficits in making social inferences with minimal context relative to controls that were independent of impairments in cold executive function and employment status.

**Table 6:** **Hierarchical multiple regression analysis of the relationships between ASD status and TASIT part 2 (social inference-minimal) controlling for Hayling, LNS and employment status (*n* = 82)**.

**Variable**	**B**	**β**	***sr*²**	***p***	**95% CI**
Constant	−5.66				[−6.95, −4.36]
**STEP 1**					
Hayling	0.125	0.38	0.13	<0.001^***^	[0.06, 0.19]
LNS	0.07	0.18	0.02	0.09	[−0.01, 0.15]
Employment^a^	−0.36	−0.13	0.01	0.21	[−0.92, 0.21]
**STEP 2**					
Hayling	0.08	0.24	0.04	0.029^*^	[0.01, 0.15]
LNS	0.05	0.12	0.01	0.23	[−0.03, 0.12]
Employment	−0.10	−0.04	0.001	0.71	[−0.66, 0.45]
ASD status^b^	0.81	0.36	0.09	0.002^**^	[0.31, 1.31]

* *p < 0.05*,

** *p < 0.01*,

*** *p < 0.001*.

a *Employment: 1, Employed/Student; 2, Unemployed*.

b *ASD status: 1, ASD; 2, Control*.

As presented in Table 7, Hayling and LNS initially explained 24.7% of the variance in TASIT Part 3 performance [*F*_(2, 79)_ = 12.93, *p* < 0.001]. When ASD status was entered in Step 2, the total variance explained significantly increased to 33.6%, *F*_(3, 78)_ = 13.15, *p* < 0.001 (adjusted *R*^2^ = 0.31). ASD status explained an additional 8.9% of the variance in TASIT Part 3 scores (1, 78) = 10.47, *p* = 0.002. In the final model, only LNS (*sr*^2^ = 0.04) and ASD (*sr*^2^ = 0.09) were significant predictors. Therefore, consistent with TASIT Parts 1 and 2, the ASD group demonstrated deficits in making social inferences with enriched context relative to controls that were independent of impairments in cold executive function.

**Table 7:** **Hierarchical multiple regression analysis of the relationship between ASD status and TASIT part 3 (social inference-enriched) controlling for the Hayling and LNS (*n* = 82)**.

**Variable**	**B**	**β**	***sr*²**	***p***	**95% CI**
Constant	−4.22				[−6.13, −2.32]
**STEP 1**					
Hayling	1.50	0.33	0.09	0.002^**^	[0.56, 2.43]
LNS	0.65	0.28	0.07	0.010^**^	[0.16, 1.13]
**STEP 2**					
Hayling	0.77	0.17	0.02	0.13	[−0.22, 1.76]
LNS	0.51	0.22	0.04	0.03^*^	[0.042, 0.98]
ASD status^a^	0.73	0.35	0.09	0.002^**^	[0.28, 1.17]

* *p < 0.05*,

** *p < 0.01*.

a *ASD status: 1, ASD; 2, Control*.

## Discussion

The broad aim of this study was to investigate the profile of hot and cold executive function impairments in high-functioning adults with ASD relative to matched controls. Overall, the ASD group demonstrated general impairments in hot and cold executive functions (i.e., emotion recognition, ToM, working memory and response initiation and suppression) when compared to matched controls. Further, the impairments in emotion recognition and ToM demonstrated by ASD participants were independent of deficits in working memory and response initiation and suppression. The pattern of findings and the theoretical and clinical implications will now be discussed.

Consistent with previous research, high-functioning individuals with ASD demonstrated deficits in working memory (Bennetto et al., 1996; Williams et al., 2005; Steele et al., 2007), response initiation and suppression (Boucher et al., 2005; Hill and Bird, 2006; Johnston et al., 2011) and multiple components of social cognition (Baron-Cohen et al., 2003; Goldenfeld et al., 2005; Auyeung et al., 2009; Baez et al., 2012; Mathersul et al., 2013). However, in contrast to the findings of Baez et al. (2012), participants with ASD in the present study were significantly poorer at recognizing emotions on TASIT Part 1 than matched controls. A likely explanation for these contrasting findings for emotion recognition relates to statistical power; namely, the sample size (*n* = 42) was larger in the present study than the study by Baez et al. (*n* = 15).

Further, analyses on TASIT Part 1 revealed that participants with ASD were significantly poorer at identifying both positive and negative emotions than controls. Interestingly, there were no significant between group differences in accuracy for the emotions of anxiety and revolted. Although there are few previous studies examining recognition of different emotions in high-functioning adults with ASD, a meta-analysis by Uljarevic and Hamilton (2013) investigated emotion recognition in 48 studies with ASD samples spanning a broad age range (note: there were no significant effects of age or IQ on emotion recognition performance). Overall, the meta-analysis yielded a large effect size which indicated a general impairment in emotion recognition. However, they found that the ability to recognize happiness was only marginally impaired, whereas impairments in fear recognition were more marked than happiness. One potential explanation for the inconsistency between these findings and that of the present study is that most studies included in the meta-analysis employed tasks involving static faces rather than dynamic stimuli. Further, in most studies reviewed in the meta-analysis, happiness was treated as a “baseline” emotion for comparison of impairment between different emotions (Uljarevic and Hamilton, 2013). Overall, the present findings support the view that high-functioning adults with ASD experience a more generalized impairment in the recognition of positive and negative emotions. However, they may find some emotions more difficult to recognize (e.g., neutral expressions) than others (e.g., revolted) in dynamic displays.

The overall finding that high-functioning individuals with ASD were significantly poorer at making social inferences than controls is in line with previous research (Perner and Wimmer, 1985; Perner et al., 1989; Happé, 1994; Baron-Cohen et al., 1997, 2003; Stone et al., 1998; Goldenfeld et al., 2005; Auyeung et al., 2009; Mathersul et al., 2013). These results add further support to the idea that contextual insensitivity (i.e., appraising critical information as unimportant while ignoring essential contextual stimuli) is a key mechanism underpinning deficits in social cognition for high-functioning individuals with ASD (Baez and Ibanez, 2014; Vermeulen, 2015). However, in contrast to Mathersul et al. (2013), who did not find a between group difference for sincere exchanges (i.e., SI-M or simple ToM judgements), the present study identified that participants with ASD were significantly poorer at perceiving sincere interactions. One potential explanation relates to the lower mean RAADS-R score (i.e., 126.4) for the sample in the study of Mathersul et al. (2013) when compared to the present sample (i.e., 137.88). Thus, more severe ASD symptoms may have contributed to the poorer performance of ASD participants in making simple ToM judgements about sincere interactions relative to controls.

The finding that ASD participants were impaired at detecting sarcasm and lies when contextual information is enriched (i.e., SI-E or advanced ToM) is consistent with previous research (Mathersul et al., 2013). However, unlike Mathersul et al. (2013), significant differences were not observed for the “doing” and “thinking” probes. A possible explanation for these results relates to the differential demands placed on executive control processes between SI-M and SI-E tasks (Castelli et al., 2002; McDonald, 2013). More specifically, to interpret dynamic social interactions, multiple sources of information need to be held online whilst integrating relevant cues (e.g., facial expression, body language, linguistic content, and context) to understand the meaning of the interaction, and formulate an appropriate and timely response. When less contextual information was provided (i.e., SI-M), and there were less demands on working memory, ASD participants in the current study were significantly poorer at answering all response probes (i.e., doing, saying, thinking, and feeling) relative to controls. When contextual information was enriched and there were greater demands on working memory to support social inference skills, the performance of ASD participants on the “doing” and “thinking” probes was more consistent with controls. Therefore, despite overall poorer performance on measures of social inference, the pattern of impairment across the response probes may have been influenced by the varying demands on cold executive function processes between the minimal and enriched conditions of SI-M and SI-E.

The independence of hot and cold executive functions and differential contributions of working memory and response initiation and suppression processes to TASIT performance was further investigated using regression analyses. Individuals with ASD demonstrated poorer performance on measures of emotion recognition and social inference than controls after controlling for cold executive functions. The findings also demonstrated that the ability to recognize emotions and make correct social inferences during dynamic displays was supported by working memory and response initiation and suppression processes, irrespective of participants' ASD status and employment status. When contextual information was minimal, better response initiation and suppression skills supported correct social inferences. Conversely, when contextual information was enriched, stronger working memory skills enhanced the ability to make correct social inferences.

Overall, the present results extend upon those of Baez et al. (2012) and support the distinction between impairments in cold and hot executive functions for adults with ASD. Further, consistent with neurophysiological evidence (Bauman, 1996; McAlonan et al., 2005; Ashwin et al., 2007; Lieberman, 2007), these findings suggest that hot and cold executive functions are supported by an integrated neural network. Hence, response initiation and suppression and working memory processes support emotion recognition and social inference skills during dynamic social interaction.

There are several study limitations that are important to acknowledge. For the ASD group, the convenience sampling method yielded a male-to-female ratio of approximately 2.8:1 and considerable variability in the age range (18–66 years). It is broadly accepted that males are more commonly diagnosed with ASD (i.e., ratio of approximately 4.3:1; (Fombonne, 2003). There was also a high proportion of individuals in the present study with elevated levels of self-reported depression, anxiety and stress symptoms and adaptive functioning was variable for the sample. More generally, given the convenience sampling approach and demographic characteristics, the participants in the present study may not be representative of the broader ASD population. Further, research is needed to examine the relationship between hot and cold executive functions, severity of ASD symptoms and psychosocial outcomes (note: a study with this objective has recently been completed by the authors and a manuscript is in preparation).

An additional study limitation was the lack of formal verification of a current ASD diagnosis from an independent clinical assessment. The reliance on a past diagnosis and self-reported symptoms may have misidentified some participants as having an ASD. Nonetheless, all participants in the ASD group reported a history of longstanding difficulties in social interaction and communication, and a restricted range of behaviors and/or interests on the RAADS-R, which is a validated assessment tool to support diagnosis of ASD (Ritvo et al., 2008, 2011). As a further limitation, the estimate of IQ was based only on Matrix Reasoning which is a non-verbal test. Although no individuals with ASD performed in the range indicating literacy difficulties on a reading test, a measure of verbal reasoning skills may have provided a more accurate reflection of verbal IQ. Related to this point, both cold executive function tests required verbal responses. Thus, it is possible that the poorer performance of the ASD sample on the cold executive function tests relative to controls may have been related to lower verbal reasoning skills.

Finally, only two tests of cold executive functions were selected for this study based on the perceived relevance of working memory and response initiation and suppression to dynamic social cognition tasks. A broader battery of tests of cold executive function (including switching, planning, reasoning and problem-solving) is needed to further determine the independence of impairments in hot and cold executive functioning for individuals with ASD in future research. The influence of other cognitive domains (e.g., language, visuo-spatial skills, and processing speed), adaptive functioning and mental health status on social cognition deficits also needs to be investigated for this population.

The present findings improve understanding of the profile of higher-order cognitive deficits underlying difficulties with social interaction for high-functioning adults with ASD. Specifically, the independence of deficits in social cognition and working memory and response initiation and suppression highlights the need for comprehensive assessment of hot and cold executive functions to aid diagnosis and inform interventions. The finding that working memory and response initiation and suppression skills contribute to the ability to recognize emotions and make social inferences suggests the likely value of targeting both hot and cold executive function impairments in interventions. There is some empirical support for the efficacy of social cognition skills training for the ASD population through computer-based programs or group-based cognitive behavioral interventions (see review by Bishop-Fitzpatrick et al., 2013). Further, Eack et al. (2013) demonstrated the feasibility, acceptability and initial efficacy of Cognitive Enhancement Therapy (Hogarty and Flesher, 1999), a comprehensive cognitive rehabilitation program, for treating impairments in “social and non-social information processing” in adults with ASD. They reported significant effects for both cognitive deficits (i.e., working memory, behavioral monitoring and perseverative errors) and social behavior. Further, research is needed to determine the efficacy of integrated cognitive rehabilitation and social cognition interventions that concurrently target cold and hot executive function deficits. It would be particularly valuable to determine whether improvements in cold executive function might positively influence improvements in hot executive functions or vice versa.

In summary, the present study identified that high-functioning adults with ASD performed more poorly on measures of both hot and cold executive functions relative to controls. Furthermore, their impairments in emotion recognition and social inference were independent of deficits in working memory and response initiation and suppression.

The finding highlight the need to assess hot and cold executive functions in clinical practice and to concurrently target impairments in both domains in interventions for the ASD population.

## Author contributions

All authors (i.e., DZ, TO, AO, and MG) made substantial contributions to the conception and design of the study. DZ and JR were involved in the recruitment of participants. DZ collected the majority of data (see acknowledgements section). DZ, TO, and MG were involved in data analysis. DZ and TO were responsible for preparing initial drafts of the manuscript. Each author was involved in drafting the work and/or critically revising it for important intellectual content and all authors gave final approval of the version to be published. All authors agree to be accountable for all aspects of the work in ensuring that questions related to the accuracy or integrity of any part of the work are appropriately investigated and resolved.

## Funding

No specific funding was utilized in conducting this study or preparing this manuscript, which was produced in partial fulfillment PhD requirements for the first author. MG is supported by a National Health and Medical Research Council of Australia Early Career Fellowship (APP1036365).

### Conflict of interest statement

The authors declare that the research was conducted in the absence of any commercial or financial relationships that could be construed as a potential conflict of interest.
